# Efficacy of Hydrochlorothiazide and low renal solute feed in Neonatal Central Diabetes Insipidus with transition to Oral Desmopressin in early infancy

**DOI:** 10.1186/1687-9856-2014-11

**Published:** 2014-06-20

**Authors:** Mary B Abraham, Shripada Rao, Glynis Price, Catherine S Choong

**Affiliations:** 1 Department of Endocrinology and Diabetes, Princess Margaret Hospital, Perth, Australia; 2 Department of Neonatology, Princess Margaret Hospital, Perth, Australia; 3 School of Paediatrics and Child Health, The University of Western Australia, Perth, Australia

**Keywords:** Neonatal Central Diabetes Insipidus, Hydrochlorothiazide, Desmopressin, Hyponatremia, Low renal solute feeds

## Abstract

**Background:**

The treatment of central diabetes insipidus (DI) with desmopressin in the neonatal period is challenging because of the significant risk of hyponatremia with this agent. The fixed anti-diuresis action of desmopressin and the obligate high fluid intake with milk feeds lead to considerable risk of water intoxication and hyponatremia. To reduce this risk, thiazide diuretics, part of the treatment of nephrogenic DI, were used in conjunction with low renal solute feed and were effective in a single case series of neonatal central DI.

**Aim:**

We evaluated the efficacy of early treatment of neonatal central DI with hydrochlorothiazide with low solute feed and investigated the clinical indicators for transition to desmopressin during infancy.

**Methods:**

A retrospective chart review was conducted at Princess Margaret Hospital, Perth of neonates diagnosed with central DI and treated with hydrochlorothiazide, between 2007 and 2013. Four newborns were identified. Mean sNa and mean change in sNa with desmopressin and hydrochlorothiazide treatment were recorded along with episodes of hyponatremia and hypernatremia. Length and weight trajectories during the first 12 months were assessed.

**Results:**

The mean change in sNa per day with hydrochlorothiazide and low renal solute feed was 2.5 - 3 mmol/L; on desmopressin treatment, the mean change in sNa was 6.8-7.9 mmol/L. There was one episode of symptomatic hyponatremia with intranasal desmopressin with no episodes of hyponatremia or hypernatremia during treatment with hydrochlorothiazide or following transition to oral desmopressin. Transition to oral desmopressin between 3 to 12 months of age was associated with good control of DI. Following introduction of solids, sNa remained stable but weight gain was slow. This improved following transition to desmopressin in one infant.

**Conclusions:**

Hydrochlorothiazide with low renal solute feed is a safe and effective treatment option in neonatal central DI. However, transition to desmopressin should be considered early in infancy following initiation of solids to facilitate growth.

## Introduction

Central Diabetes Insipidus (DI) in newborns and infants is due to the deficiency of arginine vasopressin (AVP) from the posterior pituitary and is associated with septo-optic dysplasia and other midline malformations [1]. DI should be considered in polyuric neonates with elevated serum sodium and osmolality and an inappropriately low urine osmolality (<300 mosm/kg). Polyuria is characterized by a urine volume in excess of 2 l/m^2^/24 h or approximately 150 ml/kg/24 h at birth [2]. Desmopressin acetate (1-deamino-8-D arginine vasopressin) is the long acting synthetic analogue of vasopressin, without its vasoconstrictive effect, and is the current therapeutic agent of choice in the management of patients with central DI [1]. It binds to the V2 vasopressin receptor on the distal tubules and collecting ducts of the kidney to increase water reabsorption.

Desmopressin is available in oral, nasal and parental preparations and there is a high degree of dose variability between the different forms. In general, the oral dose is about ten times greater than the intranasal dose while the SC dose is 10 times more potent than the intranasal dose [3]. There is also marked inter-individual differences in sensitivity to the drug. These factors make establishing the correct dose and frequency of administration difficult. The fixed anti-diuresis action of desmopressin and the high fluid intake necessary to meet caloric requirements of newborns with milk feeds [4] increase the risk of hyponatremia and make the management of newborns with central DI challenging and difficult.

Thiazide diuretics are used in nephrogenic DI and have been used effectively in a case series of 3 newborns with central DI when desmopressin treatment caused varying sNa levels [5]. The principal action of thiazide diuretics is at the distal convoluted tubule and inhibits the Na-Cl cotransport, inducing natriuresis. Prolonged administration leads to reduction of extracellular fluid volume with water and sodium reabsorption at the proximal tubules. This leads to reduced water and sodium delivery to the distal tubules with resultant reduction in urine output [6]. This theory only partly accounts for the reduction in urine volume. Animal models have shown that the thiazide diuretic, hydrochlorothiazide, also acts at the inner medullary collecting ducts and enhances water reabsorption by increasing osmotic and diffusional water permeability. This direct action plays an important role in reducing urine volume [7]. The antidiuretic effect of hydrochlorothiazide was more pronounced with low sodium diet with diminishing of the effect with high sodium intake [8]. The sum of dietary nitrogen, sodium, potassium, chloride and phosphorus determines the solute load to the kidneys, and, therefore, the urine osmolality [9]. Urine output increases proportionately to the renal solute load (RSL) in feeds in the presence of normal renal function and hence low renal solute feed is used concurrently with thiazides to reduce urine volume. Breast milk has the lowest renal solute load and in non-breast fed babies, commercially available formula with low renal solute load is used (Table 1) [10].

**Table 1:** Renal solute load (RSL) in feeds

**Feed**	**RSL**
	**(mOsm/L)**
Breast milk	93
Standard formula	135-260
Cow’s milk	308
Nan®HA	95

Nan®HA: commercially available formula.

The successful use of thiazide diuretics with low renal solute load was first documented in a case series of three infants with central DI [5]. However, transition from hydrochlorothiazide to desmopressin is warranted with increasing solid intake. Rivkees et al. has suggested transition at 12 to 24 months of age when infants are on at least 80% of solids for their calorie intake [4]. In the following case series of four newborns with central DI, we aim to review the efficacy of hydrochlorothiazide and low renal solute feed in the treatment of central DI by presenting the clinical response of these infants and their transition to oral desmopressin.

## Methods

The Princess Margaret Hospital Newborn Service is the sole provider of tertiary neonatal intensive care for Western Australia. There are approximately 690 admissions per year. Review of discharge diagnoses over 2007–2013 identified 4 newborns with central DI treated with hydrochlorothiazide. A retrospective chart review of these patients was conducted to characterize their clinical features and management. Diabetes insipidus was diagnosed by the presence of hypernatremia (sNa > 150 mmol/L), high serum osmolality (>300 mOsm/L) and low urine osmolality (<300 mOsm/L). The co-existence of other pituitary hormone deficiencies was also reviewed.

During the treatment period in which the newborns were either on desmopressin or hydrochlorothiazide, mean sNa (SD) and mean change in sNa (SD) per day were reviewed; the latter was calculated against time, using sodium measurements taken > 8 hours apart. Episodes of hyponatremia (sNa <130 mmol/L) and hypernatremia (sNa >150 mosm/L) were documented. The age of the infant and reason for transition from hydrochlorothiazide to oral desmopressin was also recorded. Growth parameters during the first 12 months were assessed.

Informed consent according to Institutional HREC procedures was obtained from parents for this case series.

## Results

There were 4 newborns treated for central DI with thiazide diuretics. DI was secondary to septo-optic dysplasia in 3 newborns and to holoprosencephaly in one newborn. The newborns were born at term with diabetes insipidus manifested in the first week of life. In the three newborns with septo-optic dysplasia, there were coexistent hormone deficiencies including growth hormone, thyroxine and cortisol. DI was isolated in the case (S4) with holoprosencephaly.

Intranasal desmopressin was the first drug instituted in 2 newborns. Wide fluctuations in sNa levels on desmopressin initiated a change to hydrochlorothiazide with low renal solute load feed and led to stabilisation of sNa levels. The subsequent 2 newborns with central DI were commenced on hydrochlorothiazide as the first line drug. The dose of hydrochlorothiazide was 2 to 3 mg/kg/day in two divided doses. The diuretic was used in conjunction with expressed breast milk or commercially available Nan® H.A. The response to hydrochlorothiazide, with sNa levels in normal range, was seen within the first 24 hours.

**Figure 1:**
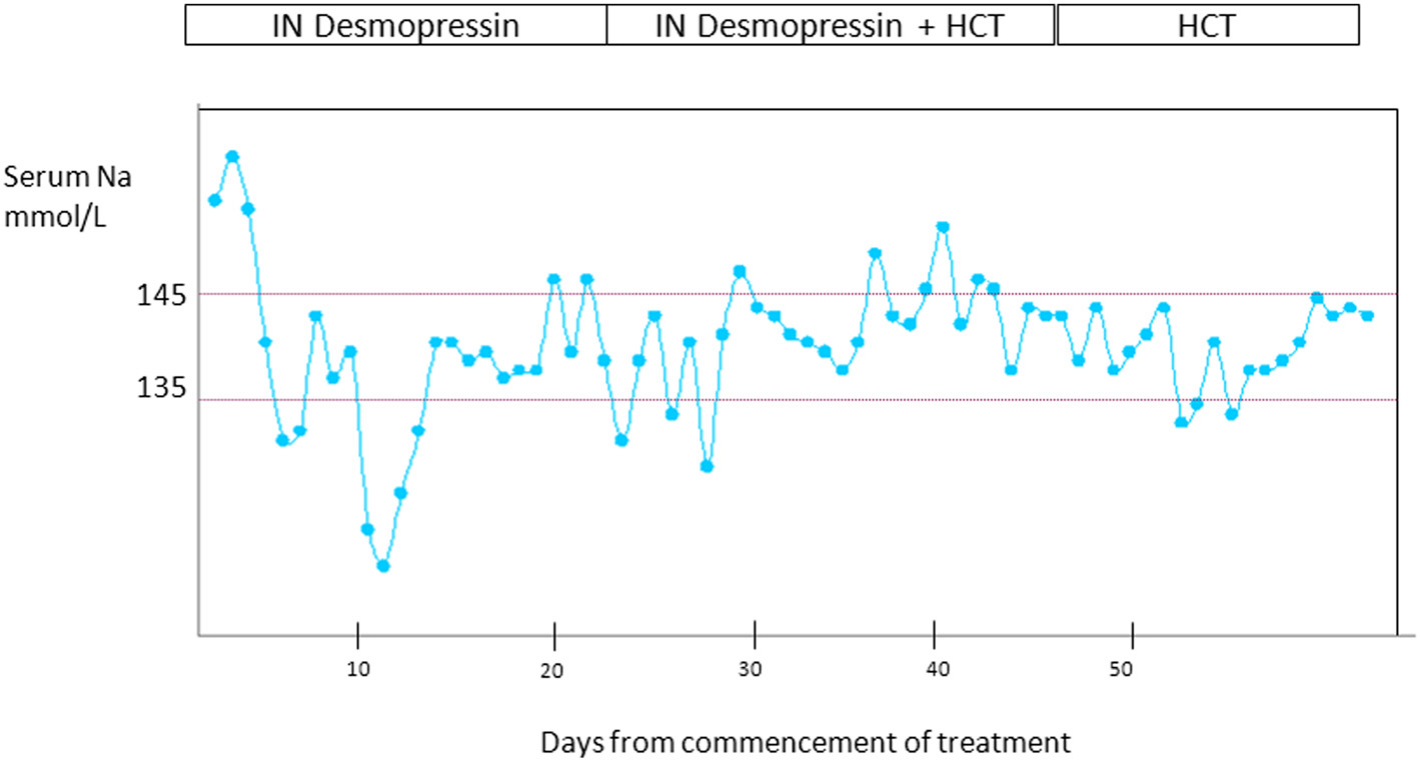
Serum Na profile in newborn with DI (S1) on intranasal desmopressin and Hydrochlorothiazide (HCT).

The mean sNa and mean change in sNa (SD) on treatment with desmopressin and hydrochlorothiazide are demonstrated in Table 2. sNa levels are more stable with hydrochlorothiazide. This is demonstrated in the sNa profile in a newborn with DI (S1) in Figure 1. Transition to oral desmopressin was initiated at 12 months of age in two infants when on significant solid intake except for S2 wherein desmopressin was commenced at 3 months of age in the background of difficult to control DI with gastroenteritis. The initial desmopressin doses ranged from 10 to 50 mcg (Table 3). There was one episode of symptomatic hyponatremia with intranasal desmopressin in the neonatal period. Importantly, there were no episodes of hyponatremia or hypernatremia during the neonatal period associated with hydrochlorothiazide and after transition to oral desmopressin. Other pituitary hormone deficiencies were carefully monitored and supplemented in the 3 cases with septo-optic dysplasia.

**Table 2:** Mean sNa and mean change in sNa (mmol/L) per day on treatment with oral desmopressin and hydrochlorothiazide (HCT)

**S**	**Mean sNa (SD)**			**Mean change in sNa (SD)**		
	**Desmopressin**	**Desmopressin (IN)+**	**HCT+**	**Desmopressin**	**Desmopressin (IN)+**	**HCT+**
	**(IN)**	**HCT**	**Low RSL**	**(IN)**	**HCT**	**Low RSL**
**1**	139	144.2	141.0	6.8	5.3	3
	+/−10.5	+/−5.4	+/−4.7	+/−5.6	+/−4	+/−2.7
**2**	142.7		143.5	7.9	-	2.7
	+/−8.5		+/−2.5	+/−6		+/−1.6
**3**			142.4		-	2.5
			+/−3.0			+/−2.4
**4**			142.2		-	2.55
			+/−2.9			+/−2.9

**Table 3:** Transition from hydrochlorothiazide to oral desmopressin

**S**	**Etiology**	**Age of transition**	**Reason for transition**	**Dose of desmopressin**	**F/U: growth**
				**(oral)**	
1	SOD	12 months	Failure to thrive	50 mcg BD	Improved growth
				10.5 mcg/kg/	
2	SOD	3 months	Acute gastroenteritis with low K; difficulty in maintaining Na	25 mcg BD	-NA*-
				4.5 mcg/kg/day	
3	SOD	6 months	Failure to thrive	10 mcg BD	No improvement in growth; concerns regarding compliance
				3.3 mcg/kg/day	
4	HPE	12 months	Failure to thrive	50 mcg BD	No improvement in growth but growth impaired due to underlying condition
			Hypernatremia	10 mcg/kg/day	

*NA: not available.

SOD: Septo-optic dysplasia.

HPE: Holoprosencephaly.

## Discussion

Desmopressin is an effective treatment for central DI; however the risk of hyponatremia is a significant adverse effect and reported for all formulations [11]. This case series demonstrates that hydrochlorothiazide combined with low solute feed resulted in less variability in sNa compared to desmopressin therapy in newborns with central DI and is a safer option in the neonatal period.

Thiazide diuretics are used in the management of nephrogenic DI [12] and their use has extended to central DI [5,13] though still not common practice. Chlorothiazide was used as the diuretic in a dose of 5 mg/kg every 12 hours and increased urinary osmolality in an infant with DI from 50 mOsm/L to 100-150 mOsm/L [5]. This modest increment in urine osmolality is a relatively large increase considering the limited neonatal renal concentrating capacity. Chlorothiazide is not licensed for use in Australia and hence we used hydrochlorothiazide at a dose of 3 mg/kg/day as used in nephrogenic DI. The drug was administered as a suspension (1 ml = 10 mg) formulated in the hospital pharmacy. Although thiazide diuretics are generally well tolerated in newborns, the clinician should be aware of the potential risk of hypokalemia which may occur during episodes of gastroenteritis. We confirm the observations of Rivkees et al. [3,4] in the case series presented here that the combined use of hydrochlorothiazide and low renal solute formula feed is effective in managing central DI whilst the patient is maintained on a liquid diet. Hydrochlorothiazide was easy to administer and did not cause any episode of hyponatremia or hypernatremia.

**Figure 2:**
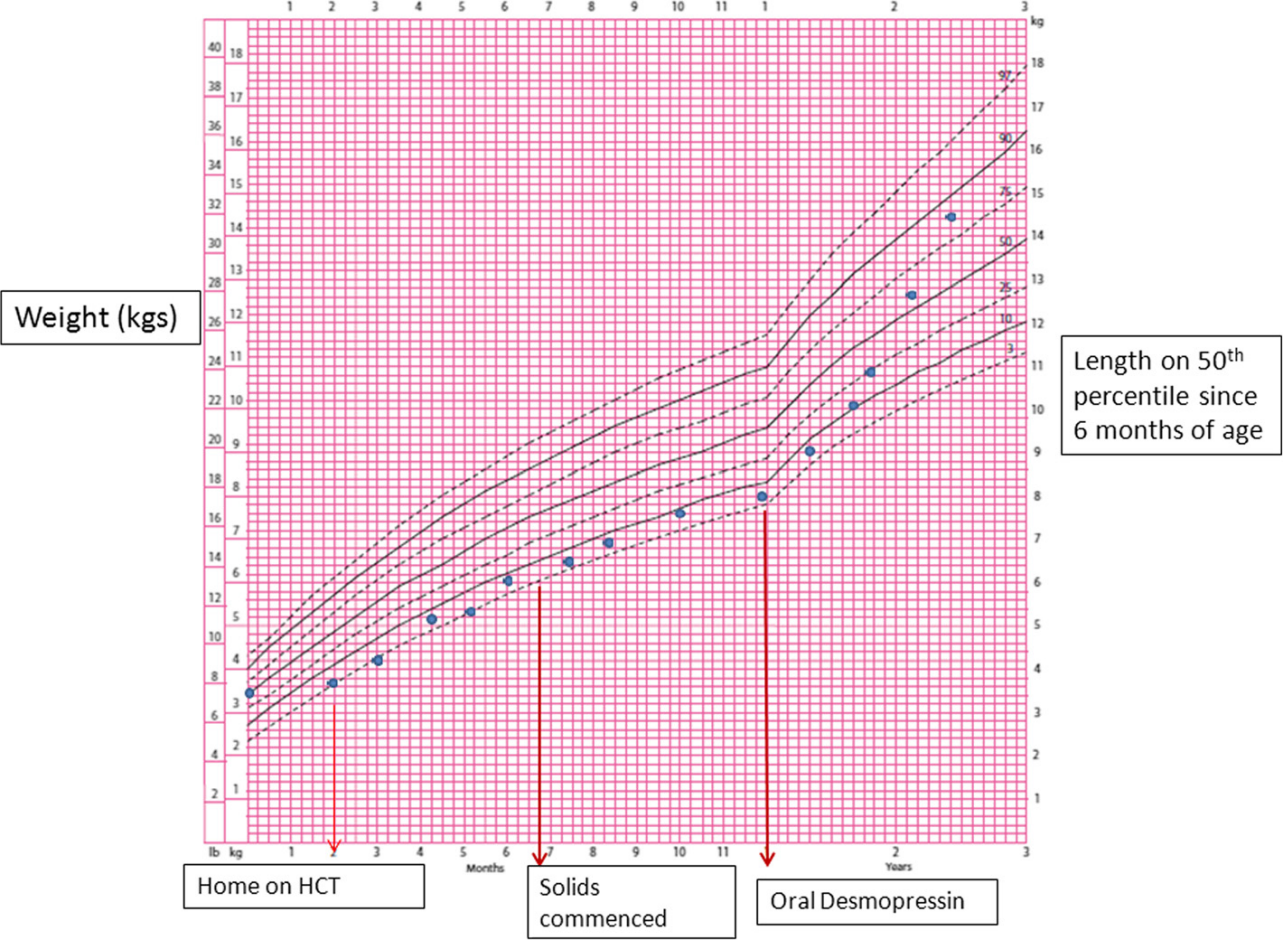
CDC growth chart of S1 demonstrates catch up growth after transition from hydrochlorothiazide (HCT) to oral desmopressin.

By 12 months, most infants have nutritious choices from the wide variety of foods eaten by the rest of the family [14] and the renal solute load will vary depending on the protein intake. If a low renal solute diet is maintained, the sNa may be stable with hydrochlorothiazide therapy as in S1 who remained on low renal solute formula until one year of age with supplemental solids. However, she failed to gain weight although her length was not compromised. Her growth improved significantly following institution of oral desmopressin as demonstrated in Figure 2. In S4, the sNa level was high at 12 months of age as she was on standard formula with no reduction in volume of feeds with minimal solid intake. We infer from both these cases that the efficacy of hydrochlorothiazide is reduced with increased renal solute load. It should be noted that growth in S4 remained poor following institution of desmopressin due to her underlying medical condition (holoprosencephaly) [15]. Based on our findings from these cases, hypernatremia is a late clinical indicator but transition should be considered at an early stage of infancy before the infants are on significant solid intake. Weight loss of 0.8 to 1.5 kg was demonstrated in 4 patients (7–17 years) with vasopressin resistant DI when commenced on hydrochlorothiazide with regain of weight on cessation of the drug [8] which the authors believe is secondary to the natriuresis induced by diuretic. Chronic diuretic therapy also causes losses of potassium, magnesium and zinc and can inhibit protein synthesis and growth [16].

Intranasal and SC desmopressin have been used in central DI in infants and recently, there is increasing use of oral desmopressin. Blanco et al. compared the sNa profile of 6 infants with SC and 4 infants with intranasal desmopressin in infants aged less than 12 months of age and found that SC desmopressin was effective and better at maintaining sNa in range than intranasal desmopressin though symptomatic hyponatremia was not observed in either group [17]. However, symptomatic hyponatremia with central pontine myelinolysis has been described in a malnourished 3 year old with holoprosencephaly on intranasal desmopressin [18]. Use of oral desmopressin during the newborn period was earlier limited to case reports as in Table 4[19-22]. An oral preparation was first used in view of difficulty with administering intranasal solutions in a patient with cleft lip/palate [20]. These first case reports were followed by a case series of infants with central DI [23]. Oral desmopressin was commenced at diagnosis (7–300 days) in 11 infants with central DI with a median initial dose of 2 mcg/kg/day (0.26 – 18.5). Oral desmopressin was found to be superior to the intranasal form with less sNa fluctuations and has been increasingly used as treatment in majority of infants and toddlers [24]. The dose of desmopressin in infancy was lower at 1.1 (0.9 – 1.4) mcg/kg/day while the median oral dose in older children was higher at 9.5 (4.2 – 17.0) mcg/kg/day. Our experience with the use of oral desmopressin outside the neonatal period has been similar with doses ranging from 3.3 to 10.5 mcg/kg/day associated with good efficacy and relatively stable sNa levels. In our centre, oral desmopressin (Minirin 200 mcg) is dissolved in 20 ml of water and the required dose is administered with a syringe. This increasing safety and efficacy data of oral desmopressin supports the early transition to oral desmopressin.

**Table 4:** Use of oral desmopressin in infants <3 months of age

**Case**	**Author**	**Etiology**	**Initial Rx**	**Age of oral desmopressin**	**Oral dose**	**Oral dose preparation**
**1**	Stick [19]	Midline defect	IN	D33	5 mcg OD increased to BD dose	Intranasal solution
**2**	Atasay [20]	Intracranial haemorrhage	IN	D73	(5 mcg/day) 2.5 μg/kg/day, twice daily	Minirin® tablet, 89 μg,
**3**	Ozaydin [21]	ECP syndrome**	Oral	< 1 month	2.5 mcg/kg/day, twice daily	Minirin® tablet, 89 μg,
**4**	Kollamparambil [22]	Transient	IV	2 months	4 mcg/day in divided doses	-NA*-

*NA: not available.

**ECP syndrome: ectrodactyly and cleft lip/palate.

## Conclusion

In conclusion, we report our experience of 4 neonates with central DI treated with hydrochlorothiazide and low solute feeds, which supports this as a safe and effective alternative to desmopressin for treatment of central DI during the neonatal period. Our results, however, suggest that hydrochlorothiazide therapy should be limited to the neonatal and first few months of age. Transition to desmopressin should occur early during infancy, at the time of initiation of solid nutrition, in order to facilitate growth.

## Competing interests

The authors declare that they have no competing interests.

## Authors’ contributions

MA collated clinical information of the cases and wrote the manuscript. SR and GP referred cases, provided clinical details, expert opinion and offered critical input. CC oversaw all aspects of the manuscript and edited the manuscript. All authors approved of the final version of the manuscript.
